# Phenotypic and genetic characterization of differential galacto-oligosaccharide utilization in *Lactobacillus plantarum*

**DOI:** 10.1038/s41598-020-78721-4

**Published:** 2020-12-10

**Authors:** Jori Fuhren, Markus Schwalbe, Lucía Peralta-Marzal, Christiane Rösch, Henk A. Schols, Michiel Kleerebezem

**Affiliations:** 1 Host Microbe Interactomics Group, Wageningen University and Research, De Elst 1, 6708 WD Wageningen, The Netherlands; 2 Laboratory of Food Chemistry, Wageningen University and Research, Bornse Weilanden 9, 6708 WG Wageningen, The Netherlands

**Keywords:** Food microbiology, Bacterial genes, Dietary carbohydrates

## Abstract

Several *Lactobacillus plantarum* strains are marketed as probiotics for their potential health benefits. Prebiotics, e.g., galacto-oligosaccharides (GOS), have the potential to selectively stimulate the growth of *L. plantarum* probiotic strains based on their phenotypic diversity in carbohydrate utilization, and thereby enhance their health promoting effects in the host in a strain-specific manner. Previously, we have shown that GOS variably promotes the strain-specific growth of *L. plantarum*. In this study we investigated this variation by molecular analysis of GOS utilization by *L. plantarum*. HPAEC-PAD analysis revealed two distinct GOS utilization phenotypes in *L. plantarum.* Linking these phenotypes to the strain-specific genotypes led to the identification of a *lac* operon encoding a β-galactosidase (*lacA*), a permease (*lacS*), and a divergently oriented regulator (*lacR*), that are predicted to be involved in the utilization of higher degree of polymerization (DP) constituents present in GOS (specifically DP of 3–4). Mutation of *lacA* and *lacS* in *L. plantarum* NC8 resulted in reduced growth on GOS, and HPAEC analysis confirmed the role of these genes in the import and utilization of higher-DP GOS constituents. Overall, the results enable the design of highly-selective synbiotic combinations of *L. plantarum* strain-specific probiotics and specific GOS-prebiotic fractions.

## Introduction

Immediately after birth the gut of infants is rapidly colonized by microbes, which marks the start of a dynamic and interactive development during the first years of human life^1^. Amongst other factors, diet can have a profound effect on the modulation of the early gut microbiota. Human milk oligosaccharides (HMO) are a structurally diverse group of carbohydrates present in human milk that are strong drivers of infant gut microbiota development, and it has been shown that in particular *Bifidobacterium* species are HMO-utilization specialists^2^. In situations where breastfeeding is not feasible, infant formula provides an alternative to mimic the nutritional composition of breastmilk^3^. Although bovine milk is mostly devoid of oligosaccharides, lactose based oligosaccharides can be produced in large quantities by incubation of various microbial β-galactosidases with high concentrations of lactose to catalyze transglycosylation reactions to produce galacto-oligosaccharides (GOS)^4^. The chemical composition of GOS components (i.e., linkage type, and degree of branching and degree of polymerization) is highly dependent of the reaction conditions, as well as the specific β-galactosidase enzyme used during production, which results in GOS preparations that are commonly a composite of multiple different oligosaccharides^5^. GOS, together with fructo-oligosaccharides (FOS), can support the early gut development in a similar fashion as HMO^6–8^, and are a continued subject of research to improve infant formula.

The application of GOS also extends to health benefits associated with its dietary-supplementation in human adults and animals that are mainly related to its bifidogenic effects^9,10^ and the stimulation of intestinal lactobacilli (and enterococci) in lactose-intolerant individuals and healthy elderly^11,12^. GOS and HMO compounds can facilitate the growth of several lactobacilli^13,14^, and various strains of different *Lactobacillus* species are marketed as probiotics, including strains of *L. rhamnosus, L. reuteri, L. casei, L. acidophilus* and *L. plantarum*^15^. Previously, we have analyzed the growth of 77 *L. plantarum* strains on a specific GOS substrate (Vivinal GOS), revealing that all strains could grow on this substrate but to a significantly different extent, indicating strain-specific variation of GOS-utilization within the *L. plantarum* species^16^. *L. plantarum* is found in a wide range of ecological habitats including silage, fermented and non-fermented food products and the gastro-intestinal tract of humans and animals, and displays a notable phenotypic and genotypic diversity, in particular in its carbohydrate metabolism repertoire^17,18^. In lactobacilli in general, it is thought that GOS is imported by lactose permeases (LacS), which was verified in *L. acidophilus* NCFM to be the sole importer for GOS and lactose^19^. Once imported, lactose is hydrolyzed by β-galactosidases of Glycoside Hydrolase family 2^20^ (LacZ/LacLM), whereas GOS is proposed to be digested by GH family 42 (LacA-type) β-galactosidases^21,22^. Not much is known about the mechanism of import and subsequent hydrolysis of GOS in *L. plantarum,* although a recent comparative transcription analysis of *L. plantarum* STIII revealed the GOS-growth induced upregulation of two gene clusters harboring various galactose- and lactose utilization genes, including *lacS, lacA, lacL and lacM*^23^. Similar genes were also identified to be involved in the utilization of GOS in *L. reuteri* ATCC PTA-6475^22^.

In this study we further explored the variable capacity of 21 *L. plantarum* strains to utilize GOS for growth and determined the strain-specific utilization pattern of specific components of GOS using high performance anion exchange with pulsed amperometric detection (HPAEC-PAD), revealing two distinct GOS utilization phenotypes. Particularly the constituents of GOS with a higher degree of polymerization (i.e., DP of 3–4) (HDP-GOS) were differentially used by a subset of strains. This distinctive phenotype could be linked to the presence of a specific gene cluster (*lacAS*, *lacR*) by comparative genomic analysis and the subsequent construction of *lacA* and *lacS* deficient derivatives allowed the confirmation of the involvement of these genes in HDP-GOS utilization in *L. plantarum*.

## Results

### Differential GOS utilization in *L. plantarum* strains

**Figure 1:**
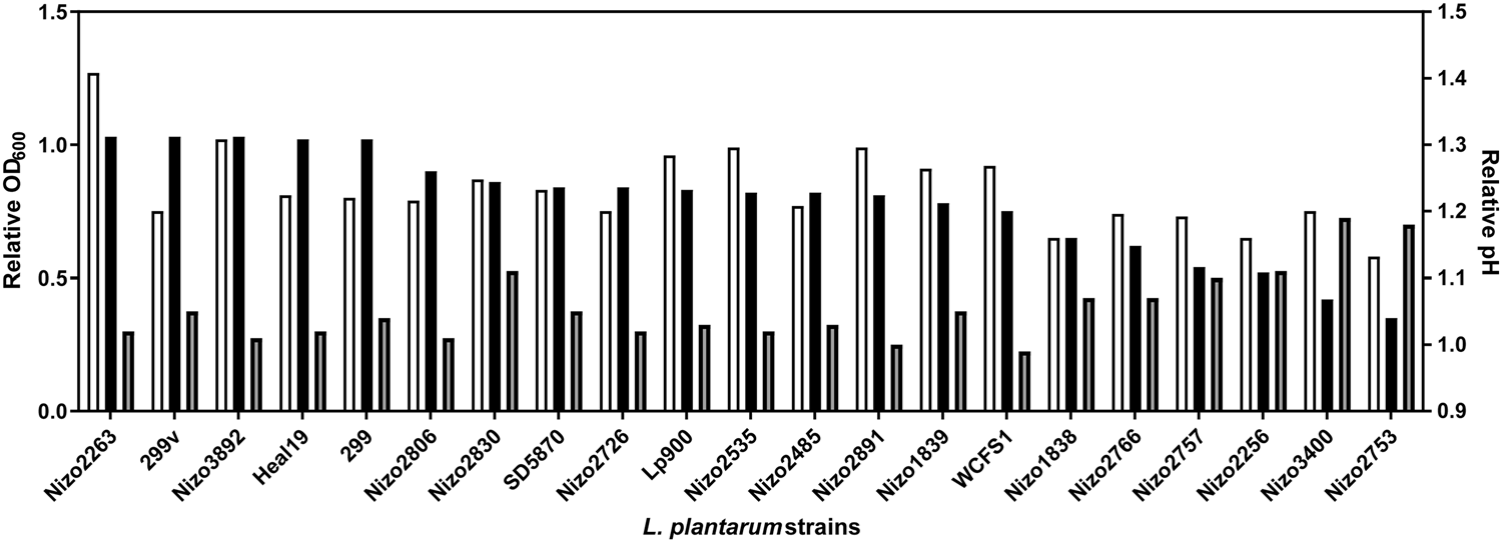
*L. plantarum* strain-specific relative growth on GOS. The relative growth (OD_600_) after 24 h of *L. plantarum* strains on ½ MRS-C supplemented with 0.5% GOS in comparison to their growth on ½ MRS-C supplemented with 0.5% glucose in batch growth experiments (OD_600_; black bars, n = 1) and pH (grey bars, n = 1) and its congruency with the relative optical density (OD_600_; white bars, n = 3) obtained in a previous reported in vitro screening^16^.

A panel of 21 *L. plantarum* strains with known genome sequences (Table 1) was selected from a larger panel of strains that was employed in an in vitro screening of strain-specific growth on Vivinal GOS containing media, to appropriately reflect the range of growth capacities observed in strains of this species^16^. These previously reported results illustrate that despite the significant variation in strain-specific growth on GOS, all *L. plantarum* strains grow relatively well on GOS-supplemented media, which was further confirmed in the present study by batch-growth experiments using optical density (OD_600_) and pH as growth reflecting read-outs (Fig. 1). The obtained strain-specific relative growth (OD_600_) of the 21 strains in GOS-supplemented medium, was significantly correlated (Pearson correlation r = 0.5967; p = 0.0043) with previously reported growth observed in the screening model (Supplemental Figure S1A). Moreover, the final pH of the same cultures was significantly correlated (Spearman correlation ρ = − 0.5381, p = 0.0119) with growth (OD_600_) (Supplemental Figure S1B). These results establish that despite the relatively moderate differences in GOS-dependent growth efficiency observed for this panel of *L. plantarum* strains these differences are reproducibly observed using different culturing conditions (96-well plate screening, versus more controlled batch cultures) and are reflected by the final pH after overnight cultivation.

**Table 1:** Strains used in this study.

Strain	Relevant feature	Reference	Source of isolation
*E. coli* NEB 5-alpha	Phage T1 resistant (*fuhA2*) DH5α™ derivative	^24^	n.a.
*L. plantarum* NC8	Plasmid free strain of *L. plantarum*	^25^	Silage
*L. plantarum* NC8Δ*lacS*	Cm^R^; Em^R^; NC8 derivative; chromosomal disruption of *lacS* by pNZ5319-*lacS-*KO integration	This study	n.a.
*L. plantarum* NC8Δ*lacA*	Cm^R^; Em^R^; NC8 derivative; chromosomal disruption of *lacA* by pNZ5319-*lacA-*KO integration	This study	n.a.
*L. plantarum* WCFS1^a^	Single-colony isolate of *L. plantarum* NCIMB8826	^26^	Human saliva
*L. plantarum* Nizo1838^a^		^18^	Human stool
*L. plantarum* Nizo1839^a^		^18^	Sour cassava
*L. plantarum* Nizo2256^a^		^18^	Human stool
*L. plantarum* Nizo2263^a^		^18^	Silage
*L. plantarum* Nizo2485^a^		^18^	Pork pickled sour Vietnam sausage
*L. plantarum* Nizo2535^a^		^18^	Fermented orange
*L. plantarum* Nizo2726^a^		^18^	Maize silage
*L. plantarum* Nizo2753^a^		^27^	Fermented sourdough
*L. plantarum* Nizo2757^a^		^18^	Fermented sourdough
*L. plantarum* Nizo2766^a^		^18^	Fermented sourdough
*L. plantarum* Nizo2806^a^		^28^	Sauerkraut
*L. plantarum* Nizo2830^a^		^18^	n.a.
*L. plantarum* Nizo2891^a^		^18^	Pickled radish
*L. plantarum* Nizo3400^a^		^18^	Milk
*L. plantarum* Nizo3892^a^		^17^	Human spinal fluid
*L. plantarum* Lp900^b^		^16^	Ogi (red sorghum)
*L. plantarum* Heal19^b^		^29^	Human GI tract
*L. plantarum* 299v^b^		^30^	Human colon
*L. plantarum* 299^b^		^30^	Human intestine
*L. plantarum* SD5870^b^		^16^	Human GI tract

^a^Nizo strain collection, ^b^Probi A/B strain collection. *n.a.* not available.

### Refinement of strain-specific utilization of GOS in *L. plantarum*

**Figure 2:**
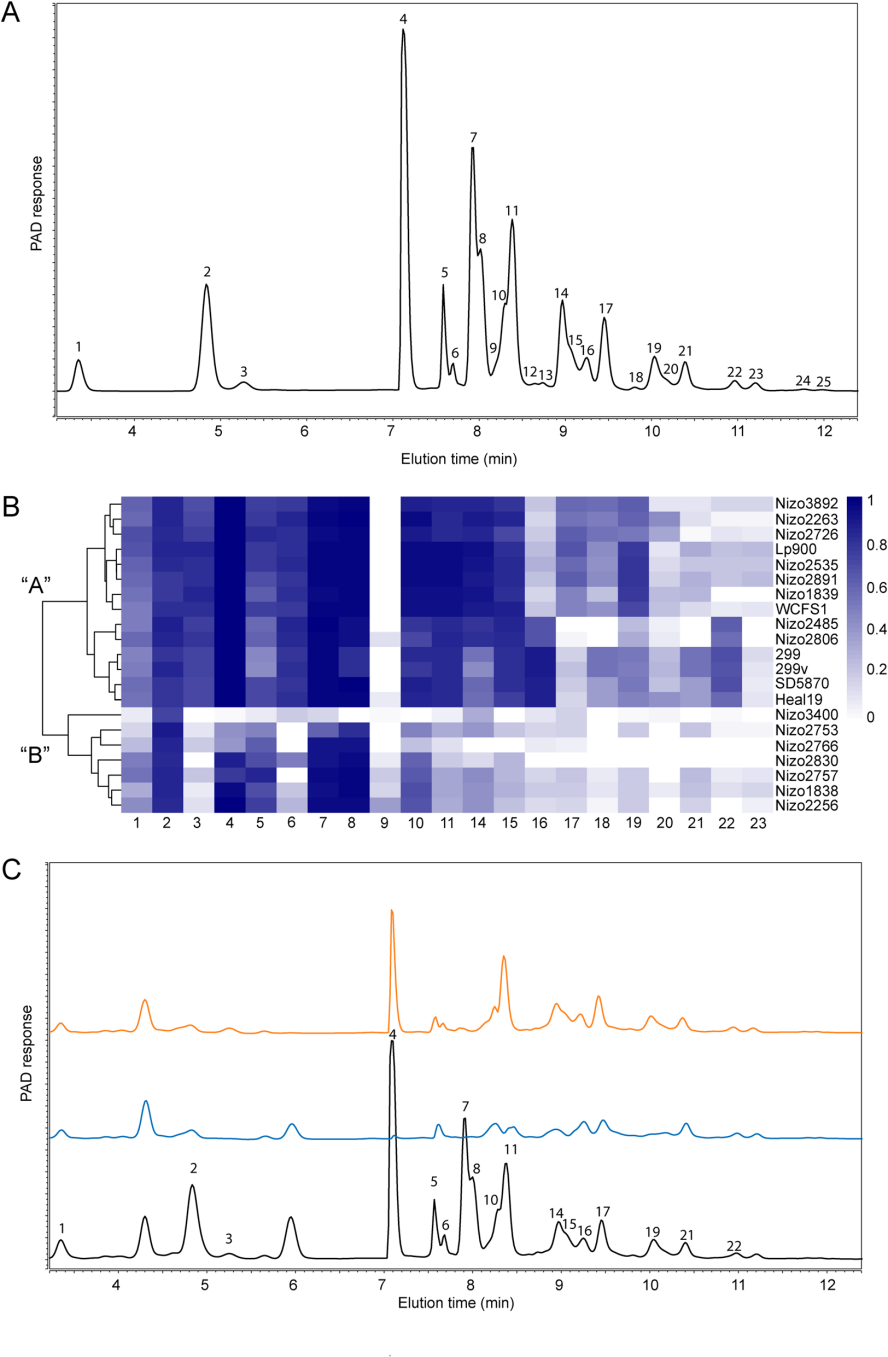
Refinement of GOS utilization by *L. plantarum* strains. (**A**) HPAEC-PAD elution pattern of the GOS substrate, in which the identified compounds include glucose and galactose (peak 2) and lactose (peak 4). See Table S1 in the supplementary material for tentative identification of the GOS peaks. (**B**) Heatmap and hierarchical clustering of relative peak surface area decrease in HPAEC-PAD response of spent culture supernatants of strains grown on 0.5% GOS compared to the original 0.5% GOS substrate after overnight incubation, with peak numbers corresponding to those presented in panel (**A**). Designated GOS utilization phenotypes “A” and “B” are represented at the first branching point of hierarchical clustering. (**C**) Exemplary HPAEC-PAD elution patterns of “A” phenotype (WCFS1, blue) and “B” phenotype (Nizo2830, orange) and the original MRS-GOS medium (black), with relevant peaks corresponding to panel (**B**).

To further explain the observed differences in relative growth performance on GOS, substrate utilization was assessed by HPAEC-PAD. The GOS used in this study is a diverse mixture of linear and branched oligosaccharides with degrees of polymerization (DP) between 2 and 8 and a multitude of β-linked glycosidic linkages, although most of the information available is limited to lower DP fractions^5^. HPAEC-PAD analysis of GOS (Fig. 2A) revealed 25 distinct peaks in an elution profile that strongly resembles a previously reported similar analysis of the same GOS product in which the chemical identity of several individual peaks was further characterized^5,31^. The similarity of the elution patterns enables the tentative identification of the majority of the separately eluting compounds (Supplemental Table S1). HPAEC-PAD elution patterns of *L. plantarum* spent culture supernatants revealed a clear distinction of two major GOS-utilization phenotypes (Fig. 2B). The majority of the strains was able to utilize the majority of the GOS substrate (14 out of 21 strains), including the oligomers of higher DP (HDP-GOS utilizers, designated phenotype “A”), whereas the remaining strains (designated phenotype “B”) only utilized lactose with variable efficiency (peak 4) and utilized a few subsequent eluting peaks that are presumed to represent β-1,2 and β-1,3 linked galactose-glucose disaccharides (peaks 7 and 8) but failed to utilize the tentative β-1,4 and β-1,6 linked galactose disaccharides (peaks 3 and 6) (Fig. 2). Additionally, a subset of strains within the “A” phenotype (i.e., strains Nizo2485, Nizo2806, 299, 299v, SD5870 and Heal19) could utilize oligosaccharides corresponding to peak 16 and 22 more efficiently than the rest of the strains displaying the “A” phenotype, but appeared to have a reduced utilization of the oligosaccharides corresponding to peak 17 and 19. Strains with the “B” phenotype displayed a relatively slow adaptation to growth on lactose, since subsequent passaging of these cultures on GOS-containing media showed accelerating rates of growth and lactose utilization but did not lead to any utilization of HDP-GOS (data not shown). To verify that the increased overall GOS utilization in “A” phenotype strains corresponded with increased growth on this substrate, the relative reduction of the cumulative surface area of all peaks in the HPAEC-PAD elution patterns per strain in comparison to the original GOS substrate was significantly correlated with the relative optical density (OD_600_) (Spearman correlation ρ: − 0.4497, p = 0.0408) and the final pH (Spearman correlation ρ: 0.6643, p = 0.0010) reached by the strains (Supplemental Figure S2). These analyses also suggested that the final pH (i.e., as a proxy for overall lactate production) provides a more accurate reflection of GOS utilization in *L. plantarum* as compared to the final optical density reached.

### Candidate *L. plantarum* genes involved in the utilization of GOS

**Figure 3:**
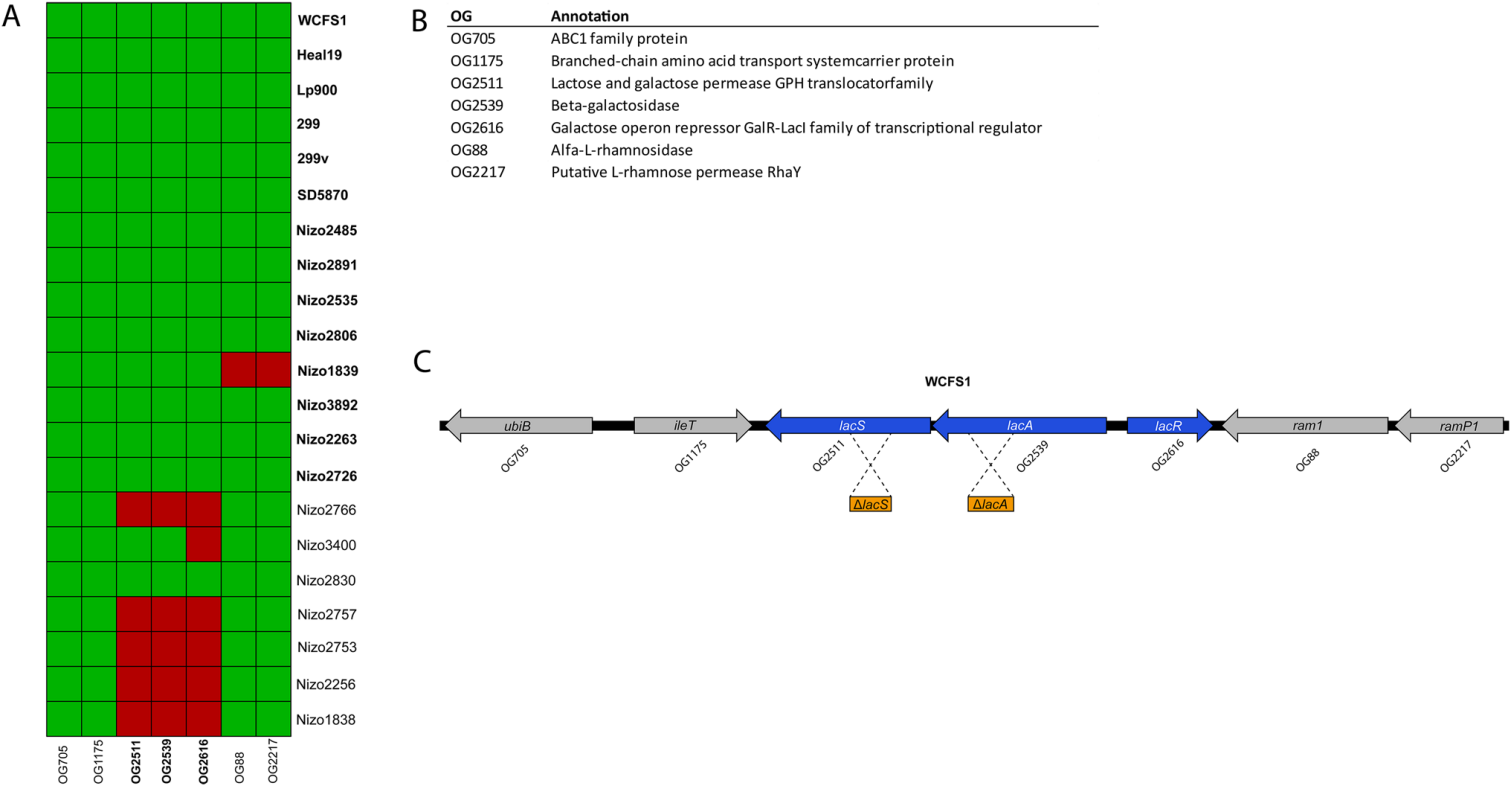
Gene cluster, identified in *L. plantarum* strains that readily utilize GOS. (**A**) Heatmap displaying the in silico gene-trait matching results based on the association between presence (green) or absence (red) of specific OGs and GOS utilization phenotypes “A” (strain names in bold) and “B” (strain names in normal font). (**B**) Predicted functions of OGs encoded in the identified operon and flanking genes in reference strain WCFS1. (**C**) Gene map of the *lac* operon and flanking genes in reference strain WCFS1 with OGs identified by gene-trait matching (blue), and genes flanking the operon (grey). The *lac* operon and flanking regions of WCFS1 are conserved in *L. plantarum* strain NC8, and homologous regions cloned in suicide vector pNZ5319 for disruption of candidate genes by single cross-over events in NC8 are presented in orange boxes for *lacS* (Δ*lacS*) and *lacA* (Δ*lacA*).

Comparative genomics to predict orthology relations between translated protein sequences of the publicly available *L. plantarum* genomes were performed to assemble an orthologous gene (OG) matrix for the subset of 21 strains using the orthAgogue tool^32^. The OG matrix was used to correlate the GOS utilization phenotypes “A” and “B” to the corresponding genotype of each strain, also known as “gene trait matching” (GTM)^33–35^, with the objective to identify candidate genes involved in the utilization of GOS and/or its breakdown products. GTM failed to identify genetic determinants that displayed a perfect presence-absence pattern corresponding with the two phenotypes within the *L. plantarum* strain panel used. However, all phenotype “A” (HDP-GOS utilizers; WCFS1, Heal19, Lp900, 299, 299v, SD5870, Nizo2485, Nizo2891, Nizo2535, Nizo206, Nizo1839, Nizo3892, Nizo2263 and Nizo2726) contain a *lac* operon consisting of OG2539 and OG2511 that are annotated as *lacA* and *lacS,* respectively, in the genome of the reference strain WCFS1. In contrast, most of the “B” phenotype strains lack this operon, albeit with the exception of the *L. plantarum* Nizo3400 and Nizo2830 strains that appeared to encode both OGs (Fig. 3). The identified genes are predicted to encode an intracellular β-galactosidase that contains a glycoside hydrolase (GH) family 42 domain (LacA), and a glycoside-pentoside-hexuronide (GPH) family lactose and galactose permease (LacS). In addition, upstream of the *lacA* gene a divergently oriented gene is located that encodes a transcriptional regulator (OG2616, annotated as *lacR* in the WCFS1 genome) that is putatively involved in the regulation of the *lacAS* operon. This divergently oriented *lacR* gene is present adjacent to the *lacAS* operon of all strains displaying the “A” phenotype, but is strikingly absent in the *lacAS* locus of the “B” phenotype strain *L. plantarum* Nizo3400. Therefore, it is tempting to speculate that *lacR* deficiency could lead to inappropriate regulation or complete lack of *lacAS* expression in this strain that underlies its failure to utilize HDP-GOS. Moreover, further analysis of the *lacAS* operon of *L. plantarum* Nizo2830 revealed that the *lacS* gene contains a disruptive mutation, rendering it a pseudogene, which explains the lack of HDP-GOS utilization in this strain. These findings provide credible explanations for the apparent genotype–phenotype discrepancy in the Nizo3400 and Nizo2830 strains, further supporting the postulated role of the *lacAS* operon in utilization of HDP-GOS compounds. Notably, gene-trait-matching analysis of the subtle difference detected between the utilization capacities observed for specific substrates (e.g., enhanced use of peak 16 and 22, and reduced use of peak 17 and 19 in strains Heal19, 299, 299v, SD570, Nizo2485 and Nizo2806) did not lead to any additional gene relationships that were considered worthwhile to pursue. Taken together, the *lacAS* operon is the prime locus associated with the difference in “A” and “B” utilization phenotypes.

### Inactivation of *lacAS* results in loss of HDP-GOS utilization in *L. plantarum* NC8

**Figure 4:**
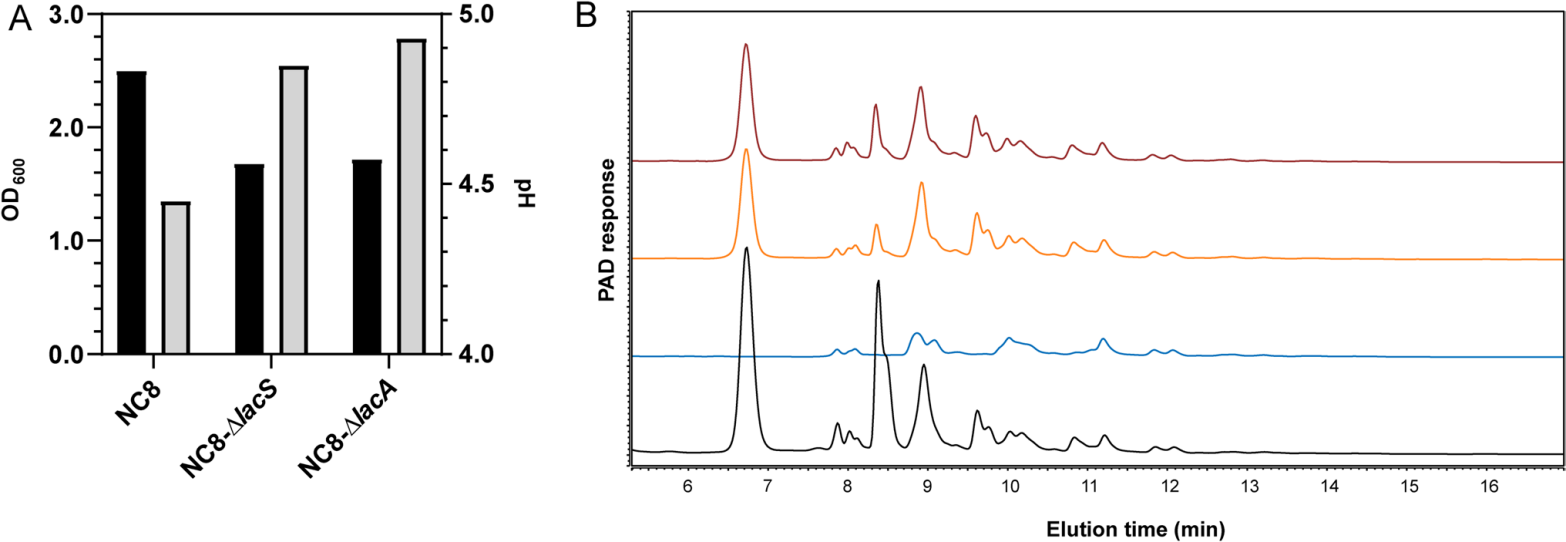
Growth of *L. plantarum* strain NC8 *lacS*- and *lacA* knockouts on GOS. (**A**) Exemplary growth characteristics of overnight cultures of *L. plantarum* NC8, NC8Δ*lacS* or NC8Δ*lacA* cultured on ½ MRS-C supplemented with 0.5% GOS with optical densities (black bars) and final pH (grey bars). (**B**) HPAEC-PAD elution patterns of MRS-C + 0.5% GOS without inoculum (black) and spent culture supernatants of NC8 (blue), NC8-Δ*lacS* (orange) and NC8-Δ*lacA* (red) grown on ½ MRS-C + 0.5% GOS.

The role of the *lacA* and *lacS* orthologues in GOS utilization was investigated in *L. plantarum* strain NC8, a plasmid free *L. plantarum* strain (Table 1). Although this strain was not part of the original set of 21 strains, based on its high efficiency in transformation it was selected as a suitable host for mutagenesis, and its chromosome encodes the *lacAS* operon. NC8 can efficiently grow on GOS as carbon substrate (OD_600_) and displays the typical HDP-GOS utilizing, “A” phenotype (Fig. 4). To construct *lacA* and *lacS* mutagenesis vectors, internal fragments of either *lacA* or *lacS* of *L. plantarum* NC8 (Fig. 3) were cloned into suicide vector pNZ5319, which contains a P_32_-*cat* cassette that confers chloramphenicol resistance when integrated into the chromosome^36^. The resulting mutagenesis vectors, designated pNZ5319-*lacA*-KO and pNZ5319-*lacS-*KO (Table 2) were transformed to *L. plantarum* NC8 and single-cross-over plasmid integration derivatives were selected and designated NC8Δ*lacA* and NC8Δ*lacS* (Table 1). The two mutant strains and their parental strain NC8, were grown overnight on GOS supplemented media and final optical densities (OD_600_) and pH were determined, demonstrating that both gene knockout strains displayed a reduction of growth and acidification as compared to the *L. plantarum* NC8 parent strain (Fig. 4). Longer incubation times (i.e., 24 h) did not lead to increased utilization. Subsequently, spent culture supernatants of these overnight cultures were analyzed by HPAEC to investigate differential substrate utilization, showing that in contrast to their parental strain both knockout strains failed to utilize the HDP-GOS constituents and displayed the typical “B” phenotype (Fig. 4). Notably, with the chosen mutation strategy for the construction of NC8Δ*lacA* it cannot be excluded that this mutant also lacks expression of the downstream *lacS* gene due to polar effects. Nevertheless, these results demonstrate that at least the LacS transport function is required for the import of HDP-GOS in *L. plantarum* NC8, and suggests that once internalized, the oligosaccharides are likely to be hydrolyzed by LacA. Finally, both NC8Δ*lacA* and NC8Δ*lacS* displayed the reduced efficiency in lactose utilization as well as the utilization of the additional GOS constituents that are typically utilized by strains of the “B” phenotype, which is apparently depends on an alternative import and intracellular hydrolysis system in *L. plantarum* (Fig. 2).

**Table 2:** Plasmids and primers used in this study.

Plasmid or primer	Relevant feature	References
**Plasmids**		
pNZ5319	Cm^R^_,_ Em^R^; pNZ5318 derivative for multiple gene replacements in gram-positive bacteria	^36^
pNZ5319-*lacA-*KO	Cm^R^_,_ Em^R^; pNZ5319 derivative containing a 612 bp region homologous to *lacA*	This study
pNZ5319-*lacS-*KO	Cm^R^_,_ Em^R^; pNZ5319 derivative containing a 522 bp region homologous to *lacS*	This study
**Primers**^a^		
S1	5′-CGTATCTCGAGAAGCTTAGTCGCAAACTGGG-3′	This study
S2	5′-CGTATGGTACCGTTACGAAAGTACGGCCCAC-3′	This study
S3	5′-CGTATGGTACCTTCACGGCTCTTGGCAGTTT-3′	This study
S4	5′-CGTATCTCGAGATTGAGCATTGACCGAGTGC-3′	This study
S5	5′-CGTATGGTACCATGCAGTTTAAATT-3′	This study
S6	5′-CGTATCTCGAGCGCGTTATCGGTCC-3′	This study
S7	5′-ATAAGGGCGGGAGCAGAATG-3′	This study
S8	5′-AATAATACGCCGCTCCCTGG-3′	This study
S9	5′-ATTCGGTCCTCGGGATATGA-3′	This study
S10	5′-CTAAAGCAATGGCACCGACG-3′	This study
S11	5′-TTGGATTTTTGTGAGCTTGGACT-3′	This study

*Cm*^*R*^ chloramphenicol resistant, *Em*^*R*^ erythromycin resistant. ^a^Underlined sequences indicate enzymatic restriction sites.

## Discussion

Various studies have demonstrated the prebiotic effect of dietary GOS to influence the host microbiome^9–12^. Especially the stimulation of endogenous *Bifidobacterium* has extensively been studied, while there are also reports on stimulation of endogenous lactobacilli^11–14^. In synbiotic applications, GOS may also selectively stimulate the in situ growth of the accompanying probiotic strain(s). Several strains of *L. plantarum* have demonstrated probiotic properties^37–40^, and their intrinsic genetic and phenotypic diversity in carbohydrate metabolism^17,18,41^ offers opportunities for selective and strain-specific stimulation by prebiotics. The GOS preparation employed here supports the growth of a large amount of *L. plantarum* strains, although this growth stimulation displays a substantial degree of variability that is reflected by the strain-specific growth stimulation detected^16^. Notably, the GOS preparation employed here contains approximately 30% mono- and di- saccharides, which tend to reduce the selectivity of growth stimulation because most bacteria can utilize these sugars. The remaining oligomeric proportion of the GOS substrate, is a complex substrate that contains a variety of oligo-saccharides that differ in glycosidic linkages and degrees of polymerization, that can be branched or linear^5^. The chemical complexity of GOS can obscure the selective growth stimulation by its specific constituents, and the analysis of the strain-specific utilization pattern of GOS that we used in this study can provide avenues towards the identification of specific GOS-constituents that allow strain-specific stimulation.

The variation in growth on GOS within individual strains of *L. plantarum* combined with HPAEC-PAD analysis of the spent culture supernatants, revealed two distinct GOS utilization phenotypes in this species. The majority of the strains, that displayed the most effective growth on GOS (i.e., “A”-phenotype) utilized the majority of the substrate, including the HDP-GOS constituents that could not be used by strains that grew less well on the GOS substrate and only appeared to utilize the lower DP constituents of the substrate (i.e., “B”-phenotype). Due to the structural complexity of GOS, it is likely that several of the peaks identified in HPAEC-PAD correspond to a combination of GOS constituents rather than a single chemical entity^5,42^. This agrees with the observation that specific compounds remain after *L. plantarum* growth that overlap with peaks in the original GOS substrate, reflecting their partial utilization. Tentative comparative analysis of the GOS elution pattern with previously reported GOS-structure analysis studies^5^ suggests that strains displaying the “A”-phenotype can utilize readily lactose as well as linear GOS up to a DP of 4, while the “B”-phenotype strains utilize only lactose and other galactose-glucose disaccharides. Moreover, peaks that are tentatively corresponding to branched GOS constituents appear not to be utilized by the “B” phenotype strains, suggesting that besides DP also saccharide structure constrains import in these strains. Interestingly, the “B”-phenotype strains also do not utilize the tentative galactose-galactose disaccharides and utilize lactose with reduced and variable efficiency relative to the “A” phenotype strains, suggesting that in these strains the pathway involved in HDP-GOS utilization also contributes to a more preferred lactose utilization. There is no correlation between the source of isolation of these strains and their capacity to utilize GOS, as both phenotypes harbor strains derived from human origin, fermented food or plant material (Table 1), confirming that the nomadic lifestyle of *L. plantarum* has consequences for the prediction of a strain’s genotype and corresponding phenotype based on the reported niche of isolation^17^. The distinction between the utilization of GOS-derived disaccharides and HDP-GOS constituents has also been reported for a panel of lactic acid bacteria strains of various species, showing that *L. acidophilus* W37 could utilize HDP-GOS, whereas *L. salivarius* W57*, L. paracasei* W20*, L. casei* W56, as well as *Pediococcus acidilactici* W143 and *Enterococcus faecium* W54 utilized only the disaccharide fraction of GOS^43^. Intriguingly, these observations are comparable to the “A” and “B” phenotype distinction observed for *L. plantarum*, illustrating that screening for prebiotic utilization should be performed at strain level, especially for species with high genetic and phenotypic diversity like *L. plantarum*.

Gene trait matching enabled the identification of the *lacAS* operon that was predicted to be involved in HDP-GOS utilization (“A” phenotype), which was confirmed in *lacA* and *lacS* deficient derivatives of *L. plantarum* NC8, which effectively led to the switching of the GOS-utilization “A” phenotype of this strain to the typical “B” phenotype. Although possible polar effects of *lacA* mutation on the downstream encoded *lacS* prevent the unambiguous confirmation of the role of the *lacA* encoded β-galactosidase in GOS utilization, the role of LacS-mediated import of HDP-GOS in *L. plantarum* is convincingly evidenced. Interestingly, inactivation of a LacS homologue in *Lactobacillus acidophilus* NCFM (~ 60% protein identity with the *lacS* of WCFS1), resulted in the complete loss of lactose, GOS and lactitol utilization in this species^19^. In contrast, the LacS deficient derivative of *L. plantarum* NC8 (NC8Δ*lacS*) still utilized lactose and several other (disaccharide) constituents of GOS, albeit with reduced efficiency. This implies that these GOS constituents are utilized by *L. plantarum* via a redundant, alternative pathway that is apparently absent in *L. acidophilus* NCFM*.* This alternative pathway in *L. plantarum* is likely to involve a lactose and galactose permease (OG2178) that is encoded in the core genome of this species (annotated as *rafP* in WCFS1), and displays 39% protein-sequence identity with both the LacS homologues of *L. plantarum* and *L. acidophilus*. Domain prediction tools suggest that OG2178 exhibits similar function as LacS, but our results indicate that its import capacity is restricted to glucose- and galactose disaccharides and that it imports lactose with reduced efficiency compared to the LacS function identified in this study. OG2178 is encoded within the core-genome encoded *gal* cluster, that encompasses three polycistronic transcriptional units associated with galactose metabolism (i.e., import, hydrolysis, Leloir pathway and transcriptional regulation)^23^. This cluster also encodes the large- (*lacL*) and small (*lacM*) β-galactosidase subunit (OG1755 and OG816, respectively) that form the heterodimer LacLM that belongs to the GH family-2 β-galactosidases, and is homologous to the *L. acidophilus* NFCM LacLM (64 and 61% protein sequence identity, respectively). Comparative transcriptome analysis in strain *L. plantarum* STIII, revealed that both the *gal* cluster and the *lacAS* operon identified in this study, were upregulated when this strain was grown on GOS as a substrate, supporting the role of both clusters in the utilization of this prebiotic substrate^23^. Interestingly, heterologous expression of the *L. plantarum* FUA3112 derived LacLM in *Lactococcus lactis* established its lactose hydrolysis activity^21^, while experiments using the purified *L. plantarum* LacLM revealed that the enzyme also has high transglycosylation activity and could be used for the synthesis of GOS di, tri- and tetra-saccharides with a high preference of β-1,6 linkages^44^. It remains to be established what the precise roles of LacA and LacLM in GOS hydrolysis are in *L. plantarum* (i.e., DP and branched or linear structure). Intriguingly, a recent investigation of GOS metabolism in *L. reuteri* ATCC PTA-6475 established a role of its LacA homologue (~ 37% identity with LacA in *L. plantarum*) in hydrolysis of galactose-galactose linkages in GOS, but also showed that this enzyme could not hydrolyze lactose, which was dependent on the LacLM homologue in this species (~ 64% identity with *L. plantarum*)^22^. Moreover, a LacS deficient *L. reuteri* derivative (~ 58% identity with *L. plantarum* LacS) displayed reduced growth efficiency on GOS as well as lactose, which is similar to the LacS mutant of *L. plantarum* in this study. Finally, analogous to *L. plantarum*, *L. reuteri* ATCC PTA-6475 encodes a redundant galactose- and lactose permease (~ 56% identity with *L. plantarum* OG2178), suggesting that in both species LacS and LacA are involved in import and hydrolysis of HDP-GOS, while lactose and other galactose-glucose disaccharides can also be imported either by LacS or the alternative permease (i.e., OG2178 in *L. plantarum*, annotated as RafP in strain WCFS1) followed by hydrolysis by LacLM.

In conclusion, our study establishes that the *L. plantarum* capacity to utilize HDP-GOS depends on the strain-specific capacity to import these substrates via LacS, while it also strongly suggests a co-dependence on the genetically linked LacA. Thereby, the presence of these genes in combination with their putative regulator LacR can accurately predict the growth capacity of *L. plantarum* strains on HDP-GOS substrates. Since commercially available GOS prebiotics commonly contain a varying fraction of mono- and di-saccharides, the distinctive phenotype among *L. plantarum* strains may be obscured in GOS-growth studies. Our study also exemplifies that much more pronounced differences in growth, and more selective and strain-specific growth stimulation may be achieved by further purification of HDP-GOS constituents, specifically those of DP3-4 since import of higher DP GOS appears to be restricted in *L. plantarum*. Moreover, detailed chemical characterization of the constituents of GOS^5,43^ may further enhance their selective application for stimulatory of growth and or activity of specific (intestinal) bacteria dependent on the substrate-specificities of their import and hydrolysis machinery. The approach taken in this study would be suitable to explore such future applications of specific fractions of GOS. In *L. plantarum* we detect only two distinctive GOS-utilization phenotypes, but this may be different in other species. In support of such possibilities, we have previously shown that the different degree of utilization of isomalto-oligosaccharides in *L. plantarum* coincides with multiple phenotype and corresponding genotype variations in specific strains of this species^16^. Finally, the results presented in this study offer opportunities for the design of strongly selective *L. plantarum* and HDP-GOS synbiotic products, aiming to synergistically enhance the health benefit to the consumer^45,46^, underpinning that deciphering the relationships between the chemical complexity of prebiotics and the selective stimulation of growth of probiotic strains by specific prebiotic constituents could enhance the health effects of synbiotics.

## Materials and methods

### Prebiotic substrate, bacterial strains, and growth conditions

Vivinal GOS was kindly provided by Friesland Campina (Domo-branch, Amersfoort, the Netherlands) and consists of 70% galacto-oligosaccharides (DP 3–8), 25% lactose and 5–6% monosaccharides glucose and galactose. The DP2–DP5 pools of GOS contain over 40 different structures^42^. The majority consists of a terminal glucose moieties elongated with β-1,6 linked galactose subunits, but also contains β-1,2, β-1,3 and β-1,6 linkages and may branched or linear. The *L. plantarum* strains used in this study were selected from the NIZO and Probi A/B strain collections (Table 1). The strains were selected from a previously studied larger panel of strains (77 strains), to reflect the range of relative growth capacities on GOS-supplemented media^16^. *L. plantarum* strains were routinely propagated in MRS medium (Merck KGaA, Darmstadt, Germany) at 37 °C without agitation. Media were supplemented with 10 μg/mL chloramphenicol when appropriate.

To verify and refine the previous results of *L. plantarum* strain-specific growth capacity on GOS as a substrate the selected strains were inoculated 1:500 from overnight cultures grown on standard MRS in 15 mL Falcon tubes (Corning, New York, USA) containing twofold diluted MRS^47^ without the addition of any carbon source (½ MRS-C) supplemented with 0.5% (w/v) GOS or glucose and grown for 24 h at 37 °C. The strain panel was also grown on ½ MRS-C without carbon source supplementation, to account for background growth on carbohydrates that are naturally present in MRS constituents. All media and GOS suspensions were autoclaved at 100 °C for 10 min. Final culture densities (OD_600_, Microplate Spectrophotometer SpectraMax M5) and final pH were determined as proxies for growth on both substrates. The relative growth capacities of the tested strains were expressed as the optical density (OD600) or pH of strains grown on medium supplemented with GOS compared to those obtained when grown on medium supplemented with glucose. Cells were removed by centrifugation (4000×g, 10 min, 4 °C) and spent culture supernatants were stored at − 20 °C for further analysis (see below).

### HPAEC-PAD peak profile characterization

Spent culture supernatants or GOS (5 mg/ml) were diluted 1:20 in MilliQ and eventual debris was removed by centrifugation (16,000×g, 15 min, room temperature), and subsequently analyzed by High-Performance Anion Exchange Chromatography with Pulsed Amperometric Detection (HPAEC-PAD) essentially as described previously^16^. Relative peak utilization per strain was estimated by determination of the area under the curve in the spent culture supernatant relative to the corresponding peaks in the original GOS substrate and was visualized in heatmaps using the pheatmap package 1.0.7^48^ in R version 3.4.0.

### Gene-trait matching

The genomes of the 21 *L. plantarum* strains for which the GOS utilization was analyzed with HPAEC-PAD, were obtained from the National Center for Biotechnology Information (NCBI) database^49^. Protein sequences were aligned with an all-versus-all bi-directional BLASTP (cut-off: E-value < 1e−05)^50^ and the resulting output was processed by OrthAgogue^32^ to cluster orthologue genes into orthologue groups (OGs). Orthologue gene families were respectively appointed as either part of the core genome or variome based on their presence in the entire- or subset of the 21 strains. An OG matrix was constructed from the assigned OGs where each OG is linked to the corresponding genome-locus tag for each of the strains, which was subsequently converted to a binary gene-presence (1) and -absence (0) matrix (Supplemental Table S2). An in silico genotype/phenotype gene-trait matching (GTM) was performed based on the generated binary matrix of orthologous genes, correlating the presence and absence of genes with the observed constituent-specific GOS utilization phenotypes (i.e., HDP-GOS utilizers [phenotype “A”] and non-utilizers [phenotype “B”]) of each strain visualized by HPAEC-PAD. Candidate OGs were ranked based on their importance (predictive value), and presence in phenotype “A” and absence in phenotype “B” to predict their correlation with the phenotype as previously described^33^. Visualization of genomic regions containing the phenotype-related candidate OGs, were performed in Artemis version 16.0.0^51^, and the gene map was created using Easyfig 2.2.2^52^, while the presence or absence of candidate OGs and their corresponding operons within the strain panel were visualized in a heatmap using pheatmap 1.0.7^48^ in R version 3.4.0. Function-annotation of candidate genes were investigated with InterProScan 5^53^ and the CazY database^20^. To predict the cellular localization of candidate OGs, the encoded proteins were analyzed with SignalP-5.0^54^, and TMHMM server v.2.0^55^.

### *LacA* and *lacS* knock-outs by homologous recombination in *L. plantarum* strain NC8

Internal fragments of the coding regions of *lacA* (612 bp, locus tag: nc8_2941) and *lacS* (522 bp; locus tag: nc8_2940) were amplified by colony PCR using a single colony of *L. plantarum* strain NC8 and primers S1-2 and S3-4, respectively (Table 2). The vector fragment of the pACYC184-derived pNZ5319 that contains a P_32_-*cat* cassette that enables selection on basis of chloramphenicol resistance^36^, was amplified by PCR using primers S5-6 (Table 2). All PCR-amplicons were purified using MSB Spin PCRapace (STRATEC Molecular, Germany), and digested with XhoI and KpnI (Thermo Fisher Scientific). The internal fragments of *lacA* and *lacS* were separately ligated with the cloning-vector fragment using T4 ligase for 90′ at room temperature (Thermo Fisher Scientific) and subsequently transformed to NEB 5-alpha competent *Escherichia coli* (New England Biolabs, Massachusetts, USA) according to the manufacturers protocol. Transformants were selected on Luria–Bertani (LB) agar plates supplemented with 10 μg/mL chloramphenicol, and colonies harboring the correct *lacA* and *lacS* mutation constructs were identified by colony PCR using primers S6 and S2 or S6 and S4 for the pNZ5319-*lacA*-KO and pNZ5319-*lacS*-KO constructs, respectively (Table 2). The selected *E. coli* clones that harbored pNZ5319-*lacA*-KO and pNZ5319-*lacS*-KO were grown overnight at 37 °C in LB media supplemented with 10 μg/mL chloramphenicol. The plasmids pNZ5319-*lacA*-KO and pNZ5319-*lacS*-KO were isolated from these overnight cultures using QIAGEN Plasmid *Plus* Midi Kit (Qiagen, Hilden, Germany) and 4 μg of the plasmids was transformed to electrocompetent *L. plantarum* NC8 using a Gene Pulser Xcell Electroporation System (Biorad, Hercules, California, USA) using a voltage of 1500 V, capacity of 25 μF and a resistance of 400 Ω. Electrocompetent *L. plantarum* NC8 cells were prepared as previously described^36^, by culturing the cells in glycine-containing media followed by several washing steps using ice-cold solution of 30% (w/v) polyethylene glycol 1450, finally using 100-fold concentrated cell-suspensions for electroporation. Electroporated *L. plantarum* NC8 cells were resuspended in MRS supplemented with 0.5 M sucrose and 0.1 M MgCl_2_ and incubated at 37 °C for 90 min, followed by plating on MRS plates containing 10 μg/mL chloramphenicol. Notably, pNZ5319-derived plasmids can replicate extrachromosomally in *E. coli*, but not in *L. plantarum*, whereby selection of chloramphenicol resistant *L. plantarum* transformants selects for a single-cross over plasmid-integration resulting from homologous recombination of the plasmid encoded internal fragment of *lacA* or *lacS* and the corresponding homologous region in the NC8 chromosome, effectively disrupting the *lacA* or *lacS* gene, respectively. Chloramphenicol resistant plasmid-integrant colonies were selected after 3 days of incubation at 37 °C and the anticipated chromosomal integration of the plasmid was verified by PCR using primers S8-9 and S10-11 for *lacA* and *lacS* integrant colonies, respectively (Table 2). Selected single colony isolates were inoculated in MRS supplemented with 10 μg/mL chloramphenicol and grown overnight at 37 °C. Overnight cultures were subcultured (1:500) in ½ MRS-C supplemented with 0.5% GOS or glucose and grown overnight at 37 °C. Optical densities of the overnight cultures were determined (OD_600_) using a Microplate Spectrophotometer SpectraMax M5 and final pH of the cultures was measured.

### Statistical analysis

To determine whether data sets were normally distributed, Shapiro–Wilk normality tests were performed with a confidence interval of 95%. Spearman and Pearson correlations were performed two-tailed with a confidence interval of 95%. All analyses were performed using GraphPad Prism version 8.00 for Windows (GraphPad Software, San Diego, California, USA).

## Supplementary Information

Supplementary Information.

Supplementary Table 2.

## Data Availability

The datasets generated during the current study are available from the corresponding author on reasonable request.
