# Supplementary material for: Phenotypic and genetic characterization of differential galacto-oligosaccharide utilization in Lactobacillus plantarum
Source: Sci Rep. 2020 Dec 10;10:21657. doi: 10.1038/s41598-020-78721-4 (PMC7728778; doi:10.1038/s41598-020-78721-4)
Supplement: Supplementary file 2 — Supplementary Table 2. [file 41598_2020_78721_MOESM2_ESM.pdf]

Supplementary Materials

Phenotypic and genetic characterization of differential galacto-oligosaccharide utilization in Lactobacillus plantarum

Jori Fuhren[1], Markus Schwalbe[1], Lucía Peralta-Marzal[1], Christiane Rösch[2], Henk A. Schols[2], Michiel Kleerebezem[1]

1: Host Microbe Interactomics Group, Wageningen University & Research, Wageningen, the Netherlands

2: Laboratory of Food Chemistry, Wageningen University & Research, Wageningen, the Netherlands

Supplementary Table S1. Tentative peak identification of Vivinal GOS based on characterization by van Leeuwen *et al* (5). See figure 2A for GOS elution pattern

Peak van Leeuwen *et al* (5)	Tentative translation to peak this study	Structure	DP[b]
na[a]	1	na	na
1	2	β-D-Gal*p* (galactose)	1
2	2	β-D-Glc*p* (glucose)	1
3	3	β-D-Gal*p*-(1→6)-D-Gal*p*	2
4	4	β-D-Gal*p*-(1→6)-D-Glc*p* (*allo*-lactose)	2
5	4	β-D-Gal*p*-(1→4)-D-Glc*p* (lactose)	2
6	5	β-D-Gal*p*-(1→4)-[β-D-Galp-(1→6)-]D-Glc*p*	3
		+ β-D-Gal*p*-(1→6)-β-D-Galp-(1→4)-D-Glc*p*	3
7	6	β-D-Gal*p*-(1→4)-D-Gal*p*	2
8a	7	β-D-Gal*p*-(1→2)-D-Glc*p*	2
8b	8	β-D-Gal*p*-(1→3)-D-Glc*p*	2
9	9	β-D-Gal*p*-(1→2)-[β-D-Gal*p*-(1→4)-]D-Glc*p*	3
10	10	β-D-Gal*p*-(1→2)-[β-D-Gal*p*-(1→6)-]D-Glc*p*	3
		+ β-D-Gal*p*-(1→3)-[β-D-Gal*p*-(1→6)-]D-Glc*p*	3
11	11	β-D-Gal*p*-(1→4)-β-D-Gal*p*-(1→4)-D-Glc*p*	3
14	12	β-D-Gal*p*-(1→4)-[β-D-Gal*p*-(1→4)-β-D-Gal*p*-(1→6)-]D-Glc*p*	4
		β-D-Gal*p*-(1→4)-β-D-Gal*p*-(1→4)-[β-D-Gal*p*-(1→6)-]D-Glc*p*	4
12	13	β-D-Gal*p*-(1→3)-β-D-Gal*p*-(1→4)-D-Glc*p*	3
13	14	β-D-Gal*p*-(1→4)-β-D-Gal*p*-(1→2)-D-Glc*p*	3
		+ β-D-Gal*p*-(1→4)-β-D-Gal*p*-(1→3)-D-Glc*p*	3
15	15	β-D-Gal*p*-(1→4)-β-D-Gal*p*-(1→2)-[β-D-Gal*p*-(1→4)-]D-Glc*p*	4
		+ β-D-Gal*p*-(1→2)-[β-D-Gal*p*-(1→4)-β-D-Gal*p*-(1→4)-]D-Glc*p*	4
16	16	β-D-Gal*p*-(1→4)-β-D-Gal*p*-(1→2)-[β-D-Gal*p*-(1→6)-]D-Glc*p*	4
		+ β-D-Gal*p*-(1→2)-[β-D-Gal*p*-(1→4)-β-D-Gal*p*-(1→6)-]D-Glc*p*	4
		+ β-D-Gal*p*-(1→4)-β-D-Gal*p*-(1→4)-β-D-Gal*p*-(1→6)-D-Glc*p*	4
17	17	β-D-Gal*p*-(1→4)-β-D-Gal*p*-(1→4)-β-D-Gal*p*-(1→4)-D-Glc*p*	4
19	18	β-D-Gal*p*-(1→4)-[β-D-Gal*p*-(1→4)-β-D-Gal*p*-(1→4)-β-D-Gal*p*-(1→6)-]D-Glc*p*	5
		+ β-D-Gal*p*-(1→4)-β-D-Gal*p*-(1→4)-β-D-Gal*p*-(1→4)-[β-D-Gal*p*-(1→6)-]D-Glc*p*	5
		+ β-D-Gal*p*-(1→4)-[β-D-Gal*p*-(1→4)-[β-D-Gal*p*-(1→4)-β-D-Gal*p*-(1→6)-]D-Glc*p*	5
18	19	β-D-Gal*p*-(1→4)-β-D-Gal*p*-(1→4)-β-D-Gal*p*-(1→2)-D-Glc*p*	4
		+ β-D-Gal*p*-(1→4)-β-D-Gal*p*-(1→4)-β-D-Gal*p*-(1→3)-D-Glc*p*	4
20	20	β-D-Gal*p*-(1→2)-[β-D-Gal*p*-(1→4)-β-D-Gal*p*-(1→4)-β-D-Gal*p*-(1→4)-]D-Glc*p*	5
		+ β-D-Gal*p*-(1→4)-β-D-Gal*p*-(1→2)-[β-D-Gal*p*-(1→4)-β-D-Gal*p*-(1→4)-]D-Glc*p*	5
		+ β-D-Gal*p*-(1→4)-β-D-Gal*p*-(1→4)-β-D-Gal*p*-(1→2)-[β-D-Gal*p*-(1→4)-]D-Glc*p*	5
21	20	β-D-Gal*p*-(1→2)-[β-D-Gal*p*-(1→4)-β-D-Gal*p*-(1→4)-β-D-Gal*p*-(1→6)-]D-Glc*p*	5
		+ β-D-Gal*p*-(1→4)-β-D-Gal*p*-(1→4)-β-D-Gal*p*-(1→2)-[β-D-Gal*p*-(1→6)-]D-Glc*p*	5
		+ β-D-Gal*p*-(1→4)-β-D-Gal*p*-(1→2)-[β-D-Gal*p*-(1→4)-β-D-Gal*p*-(1→6)-]D-Glc*p*	5
22	21	β-D-Gal*p*-(1→4)-β-D-Gal*p*-(1→4)-β-D-Gal*p*-(1→4)-β-D-Gal*p*-(1→4)-D-Glc*p*	5
23	22	β-D-Gal*p*-(1→4)-β-D-Gal*p*-(1→4)-β-D-Gal*p*-(1→4)-β-D-Gal*p*-(1→2)-D-Glc*p*	5
		+ β-D-Gal*p*-(1→4)-β-D-Gal*p*-(1→4)-β-D-Gal*p*-(1→4)-β-D-Gal*p*-(1→3)-D-Glc*p*	5
24	23	β-D-Gal*p*-(1→4)-β-D-Gal*p*-(1→4)-β-D-Gal*p*-(1→4)-β-D-Gal*p*-(1→4)-β-D-Gal*p*-(1→4)-D-Glc*p*	6
na	24	na	na
na	25	na	na

[a]: no information available, [b]: degree of polymerization

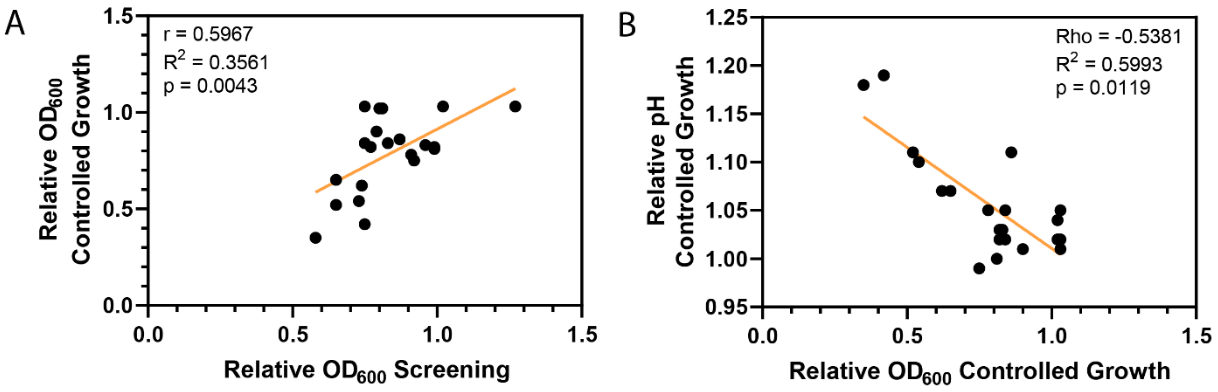

Supplemental Figure S1. Correlation analysis of screening (averages, n=3) and controlled growth experiments (n=1). A) Correlation between relative growth (OD_{600}) on GOS in 96-wells screening experiments and controlled growth experiments for the selected 21 strains. B) Correlation between relative growth (OD_{600}) and final pH for the selected 21 strains, in controlled growth experiments.

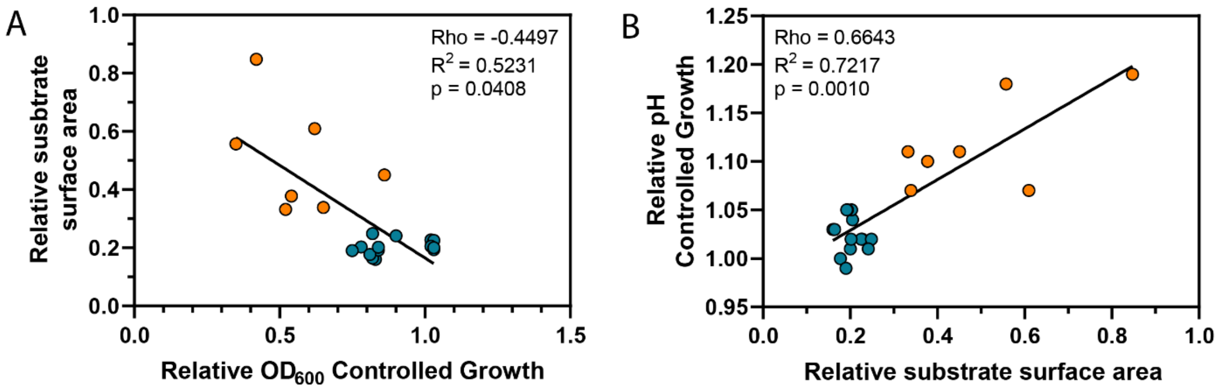

Supplemental Figure S2. Correlation analysis of surface area of HPAEC elution patterns of spent cultures supernatants of cultures grown on 0.5% GOS and their respective obtained optical density (panel A), or final pH (panel B). Designated GOS utilization phenotypes are color coded with HDP-GOS utilizers, phenotype “A” (blue) and HDP-GOS non-utilizers, “B” phenotype (orange).
